# Head elevation by 3 vs. 6 cm in ProSeal laryngeal mask airway insertion: a randomized controlled trial

**DOI:** 10.1186/s12871-016-0220-3

**Published:** 2016-08-05

**Authors:** Mi-jung Yun, Jung-Won Hwang, Sung-Hoon Kim, Hyo-Ju Hong, Young-Tae Jeon, Hee-Pyoung Park

**Affiliations:** 1 Department of Anesthesiology and Pain Medicine, National Medical Center, Seoul, Korea; 2 Department of Anesthesiology and Pain Medicine, Seoul National University Bundang Hospital, College of Medicine, Seoul National University, Seongnam, Korea; 3 Department of Anesthesiology and Pain Medicine, Seoul National University Hospital, College of Medicine, Seoul National University, Seoul, Korea

**Keywords:** Airway, PLMA, LMA, Head position

## Abstract

**Background:**

The sniffing position (neck flexion by head elevation and head extension) is commonly used for insertion of a laryngeal mask airway. However, the appropriate degrees of head elevation and head extension are unclear. In the present study, the success rate of ProSeal™ laryngeal mask airway (LMA ProSeal) insertion using two degrees of head elevation was evaluated.

**Methods:**

This prospective randomized, controlled study included 80 adult patients aged 18 to 90 years. In the 3 cm (*n* = 40) and 6 cm (*n* = 40) groups, the LMA ProSeal was inserted while the head was elevated 3 cm and 6 cm, respectively, using a pillow of the corresponding height. The success rate, and incidence of blood staining on cuff, sore throat and hoarseness were assessed. The alignments of laryngeal and oral axes were also evaluated.

**Results:**

The first attempt success rate was higher in the 3 cm than the 6 cm group (87 % vs. 60 %, *P* = 0.014). In 86 % of patients in the 6 cm group and 50 % of patients in the 3 cm group in whom the second attempt failed, the third insertion attempt was successful by using a pillow height of the opposite group. The alignments of the two axes were not different between the two groups (*P* > 0.05).

**Conclusions:**

The first attempt success rate of ProSeal laryngeal mask insertion was higher with 3 cm than 6 cm head elevation in adult patients.

**Trial registration:**

Identifiers: NCT02058030 (08/05/2015), Unique Protocol ID: phdkim1.

## Background

Much effort has focused on determining the appropriate head and neck position for successful tracheal intubation. A radiologic study [1] reported that the anatomic sniffing position (neck flexion by head elevation and head extension with a pillow) provides greater occipito-atlanto-axial extension, compared to simple head extension (head extension without a pillow), suggesting the sniffing position to be optimal for laryngoscopy during endotracheal intubation. The standard for successful endotracheal intubation is 35° of neck flexion and 15° of head extension [2]. Moreover, the head should be elevated 31–71 mm to get those angles during endotracheal intubation.

For laryngeal mask airway (LMA) insertion, it is recommended to use a pillow for neck flexion [3]; however, the appropriate degree of neck flexion is unclear. The results of clinical studies of the influence of head and neck position on the success rate of LMA insertion differ from that of tracheal intubation. The first-attempt success rate of LMA insertion using a standard position (neck fully flexed and head fully extended) or a neutral position (head extended) was investigated [4]. In that study, the insertion success rate (100 % vs. 95 %) and fiber-optic laryngeal scores were not different between the two groups.

The first-attempt success rate of ProSeal™ LMA (PLMA) insertion and fiber-optic laryngeal score according to head position (sniffing, neck flexion and head extension by means of an 8-cm-high pillow vs. head extension without a pillow) and presence of a difficult airway were assessed [5]. The two factors (head position, difficult airway) had no influence on the first-attempt success rate of PLMA insertion and fiber-optic score in that study. In most clinical situations, with the exception of patients with cervical instability, the sniffing position has been used commonly, but the appropriate degree of head elevation has not been investigated thoroughly.

The purpose of this study was to determine the pillow height (3 vs. 6 cm) that results in the highest success rate of LMA placement.

The primary outcome variable was the success rate of PLMA insertion. The secondary outcome variables were blood on the surface of the PLMA cuff, postoperative sore throat and hoarseness as indices of complications.

## Methods

### Patients and protocol

This prospective, single-center, randomized, single-blinded, parallel group comparison study was approved by the Institutional Review Board of the National Medical Center (authorization number H-1305/029-001). Written informed consents were obtained from all patients. This randomized controlled trial was registered at ClinicalTrials.gov (NCT02058030). Eighty adult patients (age range 18–90 years; American Society of Anesthesiologists physical status 1–2) scheduled for minor surgery in the supine position were enrolled. The patients were recruited from November 2013 to November 2014. Patients were excluded if they had a known or predicted difficult airway, recent sore throat, mouth opening less than 2.5 cm, or risk of aspiration (non-fasted or gastroesophageal reflux disease). Anesthesiologists who did not perform the anesthesia enrolled the participants and assigned them to one of the two groups (3 cm group, 3 cm head elevation; 6 cm group, 6 cm head elevation), using a computer-generated randomization table (generated by Mi-Jung Yun at www.randomizer.org). The allocation ratio was 1:1. The assignment was concealed in an envelope until the start of anesthesia. Both patients and evaluators were blinded to the study.

The standard anesthesia protocol was as follows: monitoring devices were connected before anesthetic induction; these included an electrocardiograph, pulse oximeter, gas analyzer and non-invasive blood pressure monitor. Anesthesia was induced with intravenous (IV) propofol (1–2 mg/kg) and inhalation of 6–8 vol% sevoflurane. Neuromuscular blockade was achieved with IV rocuronium (0.6 mg/kg). The patient’s head was elevated using a firm 3 cm pillow (3 cm group) or 6 cm pillow (6 cm group) and PLMA was inserted in the sniffing position. Anesthesia was maintained with 1.5–2.5 vol% sevoflurane in 50 % O_2_ and air. Water-based gel without a local anesthetic was applied to the posterior and lateral surface of the PLMA for lubrication, and the cuff was fully deflated before insertion.

PLMA size was determined based on age and weight. Heart rate (HR) and mean blood pressure (MBP) were recorded 1 min before and 1 min after PLMA insertion. All insertions were performed by a single experienced PLMA user who was not blinded to the pillow height. The standard insertion technique was applied in both groups using an index finger, according to the manufacturer’s instructions [3]. The patient’s head was elevated with a pillow and the head was extended using the anesthesiologist’s non-dominant hand. The index finger of the dominant hand was placed in the retaining strap of the PLMA. The PLMA was pressed against the hard palate and advanced into the hypopharynx until resistance was felt.

**Fig. 1:**
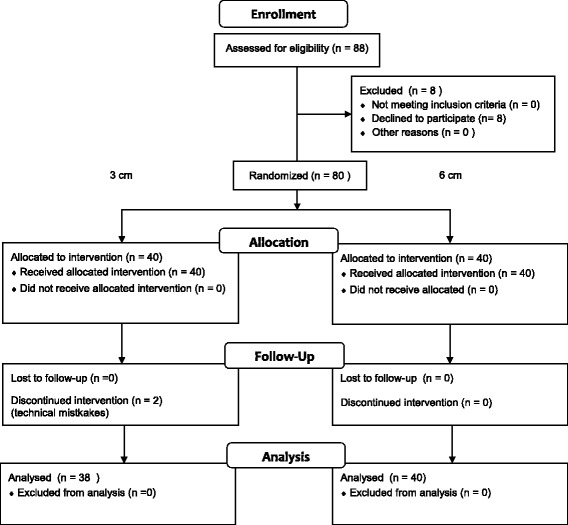
Flow diagram generated in accordance with CONSORT 2010 guidelines

The laryngeal and oral axes were measured to assess their alignment. It was postulated that insertion would be more difficult with a greater difference in angle between the two axes and the PLMA would be more likely to buckle against the posterior pharyngeal wall. The laryngeal and oral axes were assessed using images obtained on the right side of the patient during PLMA insertion. A physician, who was blinded to the study, acquired the images while the pillow was covered using a barrier. The airway axes were defined as follows: angle of the ventral neck (an imaginary line along the long axis of the trachea) and the oral axis (an imaginary midline perpendicular to the line between the upper and lower lip). The angles were measured in images of all patients. Using a protractor, each angle was assessed three times by three individual anesthesiology residents, who were blinded to the study and could not determine pillow height on the images. The difference in angle of adjacent two axes was considered the alignment between the two axes. A lesser angle difference (oral axis - ventral neck angle) indicated greater alignment of the two airway axes (Fig. 1).

The PLMA was connected to a breathing circuit after insertion, and the cuff was inflated with air until an effective airway was secured. An effective airway was defined as normal thoracoabdominal movement and a square-wave capnograph tracing. Airway pressure and end-tidal CO_2_ concentration were monitored. If insertion failed after two attempts using a pillow of the same height (head elevation), a third insertion trial was performed using a pillow height of the opposite group. If the third attempt was unsuccessful, it was regarded as a failure, and tracheal intubation was performed. The number of insertion attempts was recorded. Airway seal pressure was measured by setting the adjustable pressure limiting valve to 30 cmH_2_O and manually ventilating the patient while listening with a stethoscope over the mouth [6] and epigastrium [7] to detect oropharyngeal and gastric air leaks, respectively. The PLMA was repositioned if air leaked up the drainage tube or if ventilation was ineffective (expired tidal volume <8 ml/kg). The PLMA was removed after surgery when patients were able to breathe spontaneously and open their eyes. The following predefined complications were documented: airway reflex activation (coughing, gagging, retching, laryngospasm, bronchospasm); airway obstruction (poor air entry in the absence of upper airway reflex activation); aspiration, regurgitation, vomiting; blood staining on the PLMA surface; displacement of the PLMA from the pharynx; gastric distension (visible increase in abdominal girth with air entry into the stomach detected by stethoscopy); persistent leaks (persistent peak airway pressure < 12 cmH_2_O); hypoxia (SpO_2_ < 90 %). Sore throat and hoarseness were assessed before discharge to the ward by an anesthesia nurse, who was blinded to the study.

### Statistical analysis

Sample size was calculated based on a pilot study of 10 patients in each group. The success rate of the first attempt at PLMA insertion was 100 % in the 3 cm group and 70 % in the 6 cm group. With a 30 % difference in first attempt success being considered significant, 35 patients were required in each group, accepting a type 1 error (two-tailed) of 0.05 and a power of 90 %. An additional five patients per group were enrolled to compensate for possible loss.

Within each group, the effect on MBP and HR due to LMA insertion interaction was compared by paired *t*-test. Gender ratio, success rate, incidence of repositioning and the occurrence of complications were compared using a Fisher’s exact or chi-squared test. A value of *P* < 0.05 was considered to indicate statistical significance.

## Results

Eighty-eight patients were assessed as competent. Eight patients were excluded, and 80 participants were randomly assigned to the study groups; 40 per group. In the 3 cm group, two participants were excluded after allocation due to technical errors in PLMA insertion. The data from 38 patients in the 3 cm group, and 40 patients in the 6 cm group were subjected to statistical analysis. A patient flow diagram generated in accordance with the CONSORT 2010 statement guideline is shown in Fig. 1.

Age (59 ± 13 vs. 57 ± 13, *P* = 0.536), gender (20/18 vs. 22/18, *P* = 0.834), weight (63 ± 9 vs. 65 ± 12, *P* = 0.397), height (162 ± 7 vs. 163 ± 8, *P* = 0.391), Mallampati class (1/2/3, 31/5/2 vs. 33/5/2, *P* = 1.0) and anesthesia time (min, 73 ± 35 vs. 77 ± 33, *P* = 0.571) were similar in the two groups (Table 1). The rate of successful insertion at the first attempt was higher in the 3 cm group than the 6 cm group. However, the overall success rate, which included the first and second attempts, was not different between the two groups (Table 2). The second attempt at PLMA insertion failed in 4/38 and 7/40 patients, and the third attempt was successful in 2/4 (50 %) and 6/7 (86 %) patients, in the 3 and 6 cm groups, respectively (Table 2). The frequency of PLMA repositioning and airway sealing pressure were similar in the two groups (Table 2).

**Table 1:** Patients’ characteristics

	Group 3 cm (*n* = 38)	Group 6 cm (*n* = 40)
Age (years)	59 ± 13 (29–82)	57 ± 13 (20–77)
M/F	20/18	22/18
Weight (kg)	63 ± 9 (49–83)	65 ± 12 (42–87)
Height (cm)	162 ± 7 (148–176)	163 ± 8 (149–181)
Mallampati class (1/2/3)	31/5/2	33/5/2
Anesthesia time (min)	73 ± 35 (30–215)	77 ± 33 (35–230)

Data are presented as means ± standard deviation (range) or numbers of patients. Group 3 cm, used a 3 cm height pillow. Group 6 cm, used a 6 cm height pillow

**Table 2:** Results of ProSeal laryngeal mask airway insertion

	Group 3 cm (*n* = 38)	Group 6 cm (*n* = 40)	*P* value
No. of attempts			
1	33/38 (86)	24/40 (60)	0.014
2	1/38(3)	9/40 (23)	
Failure	4/38 (11)	7 (18)	
3	2/4 (50)	6/7 (86)	
Repositioning	3 (7)	4 (10)	0.745
Seal pressure (cmH_2_O)	26 ± 5 (15–38)	26 ± 5 (14–32)	0.799
Size of PLMA (4/5)	19/19	20/20	1.000

Data are numbers (%), means ± standard deviation (range) or numbers of patients. Group 3 cm, used a 3 cm height pillow. Group 6 cm, used a 6 cm height pillow. PLMA, ProSeal laryngeal mask airway

The angles from a horizontal line to the laryngeal and oral axes were greater in the 6 cm group than the 3 cm group, but the angles between the two axes were similar in the two groups (Table 3). The MBP and HR before and after PLMA insertion were similar in both groups (Table 4).

**Table 3:** Airway axis and alignment

	Group 3 cm (*n* = 38)	Group 6 cm (*n* = 40)	*P* value
Angle of ventral neck (°)	26.1 ± 7.6	33.7 ± 8.8	<0.01
Oral axis	90.3 ± 8.5	101.9 ± 9.6	<0.01
Oral axis - angle of ventral neck	64.1 ± 11.9	68.2 ± 13.0	0.123

Data are means ± standard deviation. Group 3 cm, used a 3 cm height pillow. Group 6 cm, used a 6 cm height pillow. Angle of ventral neck, an imaginary line along the long axis of the trachea; oral axis, an imaginary midline perpendicular to the line between the upper and lower lip

**Table 4:** Hemodynamic variables and complications

	Group 3 cm (*n* = 38)	Group 6 cm (*n* = 40)	*P* value
Mean blood pressure (mmHg)			
Preinsertion	65 ± 10	64 ± 13	0.707
Postinsertion	69 ± 14	69 ± 15	0.987
Heart rate (bpm)			
Preinsertion	68 ± 14	67 ± 15	0.731
Postinsertion	69 ± 14	70 ± 14	0.830
Blood staining on PLMA	5 (13)	6 (15)	1.000
Sore throat after recovery	10 (26)	14 (35)	0.467
Hoarseness after recovery	4 (10)	4 (10)	1.000

Data are means ± standard deviation or numbers of patients (%). Group 3 cm, used a 3 cm height pillow. Group 6 cm, used a 6 cm height pillow. PLMA, ProSeal laryngeal mask airway

There was no airway reflex activation, obstruction, aspiration, displacement of PLMA from pharynx, gastric distension, persistent leaks or hypoxia. The incidences of complications were similar in the two groups (Table 4). There was no episode of laryngospasm or respiratory distress during anesthesia or the recovery period in both groups.

## Discussion

The aim of this study was to evaluate the success rate of PLMA insertion using two degrees of neck flexion. The success rate of the first insertion attempt was higher in the 3 cm group than the 6 cm group, although the alignments of the two axes were similar between the two groups. The higher head elevation did not improve alignment of the two axes compared to the lower head elevation. In the 6 cm group, the first attempt at PLMA insertion failed in 16/40 (40 %) patients, and the anesthesiologist who performed PLMA insertion reported limited head extension in those 16 patients. The third attempt at PLMA insertion was successful in 6/7 (86 %) patients when a 3 cm pillow was used.

An appropriate degree of neck flexion is defined as that which aligns the laryngeal, pharyngeal and oral axes close to a straight line without limiting head extension.

In current clinical practice, the sniffing position (neck flexion by head elevation and head extension) is used commonly for LMA insertion, with the exception of in patients with cervical instability. The optimal degrees of neck flexion and head extension for the sniffing position during LMA insertion differ from those for tracheal intubation.

The main cause of upper airway obstruction is not the tongue but rather the epiglottis, and that head elevation with an 8-cm-high pillow raised the epiglottis from the posterior pharyngeal wall and opened the upper airway in an unconscious patient breathing spontaneously under deep halothane anaesthesia [8]. Head elevation by 8–10 cm would facilitate passage of an endotracheal tube through the vocal cords by aligning the laryngeal, pharyngeal and oral axes during conventional intubation [9].

A study of direct laryngoscopic view according to pillow height (0, 3, 6 and 9 cm) reported that the laryngoscopic view was superior with the 9 cm pillow during direct laryngoscopy in the sniffing position [10]. An adequate laryngeal view is important for successful insertion of an endotracheal tube through the vocal cord aperture. However, for PLMA insertion, smooth advancement of the PLMA (which is thicker and shorter than an endotracheal tube) through the oral, pharyngeal and laryngeal area is more important than a superior laryngeal view.

Normal mouth opening and smooth advancement of PLMA through the oral and pharyngeal cavity without impacting the posterior pharyngeal wall is an important first step for successful PLMA insertion; this could be facilitated by appropriate head extension.

Few clinical studies of the appropriate degree of neck flexion by head elevation and head extension have been performed, although LMA insertion is frequently performed by emergency physicians, anesthesiologists or laryngologists to secure the airways under diverse clinical conditions. The results of the present study suggest that the appropriate head position to facilitate LMA insertion is achieved by 3 cm head elevation, which facilitates successful LMA placement and rapid ventilation in emergency situations.

The present study had several limitations. Firstly, only two degrees of head elevation were evaluated. Pillow heights used commonly in our hospital were selected. Secondly, the pharyngeal axis was not measured in the present study because it was difficult to define the axis on images. Measurement of the pharyngeal axis on sagittal view MRI would be relevant as the PLMA is a supraglottic device, but it was difficult to obtain an MRI in every patient. Lastly, the anesthesiologist who performed PLMA insertion could not be blinded, which might have caused some bias in the results.

## Conclusion

The first-attempt success rate of PLMA insertion was higher with 3 cm than 6 cm head elevation in adult patients. Head elevation of 3 cm should be used for successful LMA placement at the first attempt.

## Abbreviations

LMA, laryngeal mask; PLMA, ProSeal laryngeal mask
